# Body Mass Index, Quality of Life and Activity Limitation Trajectories over 2 Years in Patients with Knee or Hip Osteoarthritis: A Dual Trajectory Approach Based on 4265 Patients Included in the AktivA Quality Register

**DOI:** 10.3390/jcm12227094

**Published:** 2023-11-14

**Authors:** Merete Aarsland Fosdahl, Bjørnar Berg, May Arna Risberg, Britt Elin Øiestad, Inger Holm

**Affiliations:** 1 Division of Orthopaedic Surgery, Oslo University Hospital, 0424 Oslo, Norwaymayar@nih.no (M.A.R.); 2 Department of Clinical Neurosciences for Children, Division of Pediatric and Adolescent Medicine, Oslo University Hospital, 0424 Oslo, Norway; 3 Centre for Intelligent Musculoskeletal Health, Faculty of Health Science, Oslo Metropolitan University, 0130 Oslo, Norway; bjornarb@oslomet.no; 4 Department of Sports Medicine, Norwegian School of Sport Sciences, 0863 Oslo, Norway; 5 Department of Rehabilitation Science and Health Technology, Oslo Metropolitan University, 0130 Oslo, Norway; brielo@oslomet.no; 6 Department of Interdisciplinary Health Sciences, Medical Faculty, University of Oslo, 0318 Oslo, Norway

**Keywords:** osteoarthritis, patient-reported outcomes, BMI, quality of life, activity limitations, trajectories

## Abstract

(1) Background: Recent studies claim that weight-neutral approaches emphasizing physical activity might be as effective as weight-loss-centered approaches for improving pain and physical function in patients with knee and hip osteoarthritis. The objectives were to identify distinctive groups of individuals with similar BMI, quality of life and activity limitation trajectories over two years, to compare the overall differences between BMI trajectory groups for baseline variables and to explore the probabilities of the quality of life and activity limitation trajectory groups conditional on the BMI group. (2) Methods: Baseline data for age, gender, BMI, quality of life, activity limitations, pain, general health, knee or hip osteoarthritis and follow-up data on BMI, quality of life and activity limitations at 3, 12 and 24 months were retrieved from the “Active with osteoarthritis” (AktivA) electronic quality register. Group-based trajectory modeling was used to identify distinct trajectories for BMI, quality of life and activity limitations. (3) Results: 4265 patients were included in the study. Four distinct BMI trajectories were identified, normal weight (31%), slightly overweight (43%), overweight (20%) and obese (6%). At baseline, there were highly significant differences between all BMI groups, pain increased and age and general health decreased with higher BMI. Irrespective of weight category, minimal changes in BMI were found over the two-year follow-up period. Over 80% of the participants showed moderate-to-considerable improvements both in quality of life and activity limitations. (4) Conclusions: Almost 70% of the participants belonged to the overweight trajectories. Despite no significant weight reduction over the two years, eight in every 10 participants improved their quality of life and reduced their activity limitations after participating in the AktivA program.

## 1. Introduction

Osteoarthritis (OA) is a multi-joint disease, with the knee and hip as the two most affected joints [1]. International guidelines for the treatment of hip and knee OA recommend patient education, exercises/physical activity and weight control as first-line treatments [2,3]. The guidelines recommend exercise interventions for relieving pain, improving function and quality of life and lifestyle changes to reduce body weight where needed. However, large individual treatment responses and only moderate effect sizes have been documented for different treatment approaches [4,5,6]. A high body mass index (BMI) has shown to be a significant risk factor for non-communicable diseases in general and OA in particular [7,8]. The prevalence of individuals who are overweight is particularly high in the middle-aged and older population, and weight-loss interventions are strongly recommended [9]. Thus, weight control and weight reduction are important parts of the first-line treatment in overweight and obese patients [10]. Weight control, exercises and physical activity are recommended to prevent and manage most chronic diseases, like diabetes, cancer, cardiovascular diseases and OA [11].

The impact of weight-loss programs on pain and disabilities in OA is unclear. Weight-loss strategies require permanent lifestyle changes, but, irrespective of the BMI category, we lack knowledge about the influence on pain and function, especially in a long-term perspective. A systematic review and meta-analysis concluded that patients with knee OA should be encouraged to reduce their body weight, by at least 5% within a 20-week period, to experience symptomatic relief [12]; however, knowledge about the effect in the long term is lacking.

Solanki et al. stated that prevention of weight gain might be a more realistic way to reduce the burden of the OA condition [13]. A recent literature review [14] showed that weight-loss interventions were not more effective than exercise-only interventions for knee OA. The authors suggested that guidelines should reflect uncertainty in the effect of weight-loss only interventions for reducing pain and disability [14]. Despite the uncertainty of whether the effects of exercises and physical activities on pain and function in OA are equally favorable for people with and without overweight and obesity, the updated guidelines still recommend dietary weight management as one core modality [2,3]. In a recent systematic review of clinical guidelines, Gibbs et al. state that there is a lack of specific evidence for weight-loss management in hip osteoarthritis [15]. There are still disagreements about if weight loss should be a crucial component for success in reducing pain and improving function and the quality of life in OA patients. Knowledge about the exact impact of BMI on changes in symptoms, function and quality of life in patients who have participated in management programs over time is lacking and needs to be addressed.

In a recent systematic review and individual data meta-analysis, Holden et al. aimed to identify individual baseline moderators of the effect of therapeutic exercise for reducing pain and improving physical function [16]. A total of 4241 participants were included in the analysis and a small, positive effect was found. Patients with higher pain severity and poorer physical function at baseline benefited the most from therapeutic exercises. They concluded that targeting individuals with more pronounced pain and disability might be of merit [16].

AktivA (Active with osteoArthritis) is a national implementation program, based on international guidelines including patient education, exercises and life-style changes as a first-line treatment, and it provides a stratified treatment for each patient [17].

Based on data retrieved from the AktivA quality register, the objectives of the present study were: (1) to identify distinctive groups of individuals with similar BMI, quality of life and functional ability over two years, (2) to compare the overall differences between BMI trajectory groups for baseline variables and (3) explore the probabilities of the quality of life and activity limitation trajectory groups conditional on the BMI group.

## 2. Material and Methods

### 2.1. Study Design

The present study is based on data retrieved from the AktivA quality register in Norway.

All patients signed a written consent before they completed the electronic questionnaires. The Regional Committee for medical and health research ethics approved that the data could be used in research (2018/1572/REK). A license to collect and process the data was given from The Norwegian Data Protection Authority Inspectorate (46701/4/LT).

#### 2.1.1. Inclusion Criteria

Patients were included between 2016 and 2021. They were aged ≥35 years and either had clinical symptoms compatible with degenerative meniscal tears or knee or hip OA. The diagnosis was verified by clinical examination alone, based on the EULAR evidence-based recommendations for the diagnosis of mild/moderate OA: pain, stiffness and activity limitations [18]. Radiographic verifications (X-ray, CT or MR) were not necessary, as in Norway, the restricted use of radiological examinations is strongly highlighted.

#### 2.1.2. The AktivA Implementation Program

The register includes data from the AktivA implementation program designed based on international guidelines for providing optimal habit changing strategies for patients with OA [17]. The program includes three modules: a physiotherapy certification course, a patient education and exercise program and an electronic quality register. The patient education program includes an OA class instruction course for 3 h where patients learn about symptoms and signs of OA, risk factors, treatment options, effects of physical activity and exercises and self-management skills. It also includes information about the impact of diet changes and weight loss, a healthy diet composition and lifestyle and the influence of being overweight on all-cause mortality and OA development. Potential overweight participants are recommended to consult a nutritionist for an individual diet and weight-loss counseling.

The supervised exercise program includes 2 supervised sessions per week and lasts for 6 to 12 weeks (depending on the patients’ previous experience and needs). The program is individually tailored based on the patient’s physical function and intentions and aims to give the patients knowledge and tools to change their attitudes and behavior concerning exercises and physical activity on a permanent basis. The main goal is that the patient has acquired enough knowledge and skills to continue physical activity and exercises on their own. However, we experience that providing the patients some booster sessions later on to adjust the program will give them another go.

The AktivA electronic quality register contains both physiotherapist-reported data and patient-reported data. At the 3-month follow-up, the physiotherapists report the number of supervised exercise sessions the patients have participated in at their clinic. At 3, 12 and 24 months the patients receive online questionnaires about pain and function, satisfaction with the program and how frequently they use what they have learned. Non-responders receive a reminder after two weeks.

### 2.2. Baseline Characteristics

The demographic variables included in the present study were age, gender, height, body weight, sick leave and main joint localization (hip or knee OA). Average hip/knee joint pain intensity during the last month was registered on a numeric rating scale (NRS (0 = no pain, 10 = worst pain). To provide information about the participants’ current health status, the Euro Quality of life-5D Visual Analogue Scale (EQ VAS) (0 = the worst health you can imagine, 100 = the best health you can imagine) was used [19].

### 2.3. Outcome Measures

BMI (BMI = body weight/(height × height) was estimated based on self-reported body weight at baseline, 3, 12 and 24 months, respectively, divided by the self-reported height registered at baseline.

For disease-specific quality of life, the Quality of Life (Quality of life) subscales from the Hip injury and Osteoarthritis Outcome Score (HOOS) [20] and the Knee injury and Osteoarthritis Outcome Score (KOOS) [21] were used. The scales have a range from 0 (worst) to 100 (best).

Activity limitations were measured using the Patient-Specific Functional Scale (PSFS) [22]. The questionnaire has no predefined items and was used to identify important activities that the patient can only perform with difficulties. The PSFS was developed in 1995 and elicits and records patients’ views regarding disabilities or activity limitations and identifies and evaluates relevant personal issues [23]. One to three self-selected daily life- or sports-activities which, at baseline, are impossible/difficult for the patient to perform as a result of their OA problems, and which were important for them to either improve or relearn, were identified. For the present analysis, only the first activity the patients had entered was included. The scale is an 11-point NRS (0 = unable to perform the activity, 10 = no problem to perform the activity). The instrument was also used as a part of the baseline assessment to set goals for the intervention period, to adjust the exercise program and treatment plan and to evaluate changes in activity limitations over time [17]. At 3, 12 and 24 months, the answer from the patient’s individual selected activity chosen at baseline automatically popped up in the questionnaire.

### 2.4. Statistical Analysis

Descriptive statistics were calculated to characterize the sample at baseline, both for the total population and stratified by BMI trajectory groups. A one-way between-groups analysis was conducted to explore the overall differences between trajectory groups for the baseline variables. Post hoc comparisons using the Tukey HSD were used to explore where the differences between the groups occur.

We used group-based trajectory modeling to identify distinct trajectories for BMI, quality of life and activity limitations [24]. The best univariate model was found for each outcome using a two-step model selection process to determine the optimal number of groups and shape of each trajectory. First, we changed the number of groups and repeated the analyses. Second, analyses were repeated by changing the order of the polynomial. The fit of the model was evaluated in each step, indicated by the Bayesian information criteria (BIC). The BIC balances improvements in model likelihood with the number of parameters estimated. Higher BIC values indicate a better model fit. However, we also required that the smallest trajectory consisted of 5% or more of the sample as the BIC does not always clearly identify the optimal number of groups. Censor normal specifications were used [25]. Model adequacy was assessed by the average posterior probability of group membership, which is a measure of an individual’s probability of belonging to a specific trajectory. We also assessed the correspondence between the estimated probability of group membership and the proportion assigned to each trajectory [25].

Dual trajectory modeling was used to examine the association between BMI trajectories and (1) quality of life trajectories and (2) activity limitation (PSFS) trajectories, by linking conditional probabilities across trajectory groups. We estimated the probability of each quality of life and activity limitation trajectory, conditional on membership for a given BMI trajectory. The Proc Traj plugin for Stata (StataCorp LLC, College Station, TX, USA) was used to fit the trajectory models [26].

Sensitivity analyses were performed to examine the impact of missing data, by excluding participants with missing outcome data at two or three time points.

## 3. Results

Data from 4265 participants were included in the analysis. The response rates were 84%, 61% and 53% at 3, 12 and 24 months, respectively. Table 1 presents demographic baseline characteristics for the included participants. The most frequent activity limitations which were impossible/difficult for the patient to perform were daily activities like bending, lifting and carrying (40%), walking/hiking (32%) and jogging/running (19.5%).

**Figure 1:**
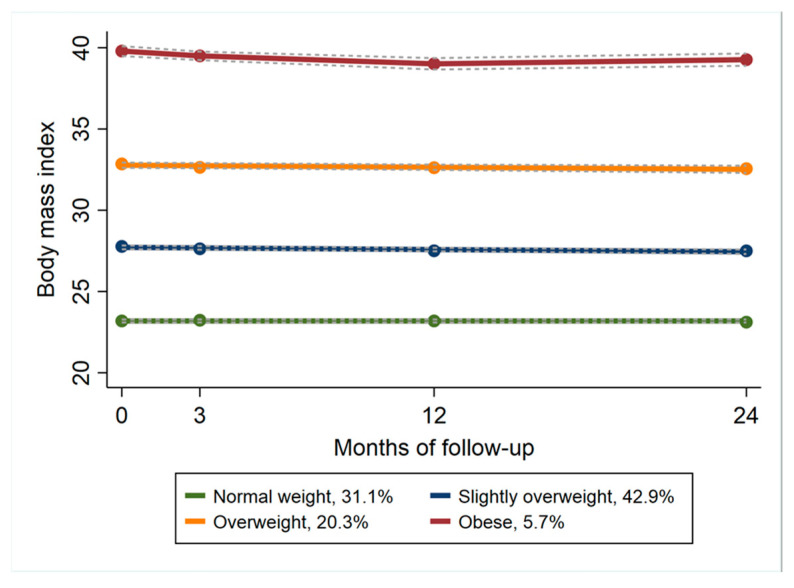
Group-based trajectories for body mass index. Each point represents the mean value for each trajectory. The solid line depicts the predicted trajectory, and the short-dashed lines represent 95% confidence intervals. The labels give the proportion of participants assigned to each trajectory.

**Figure 2:**
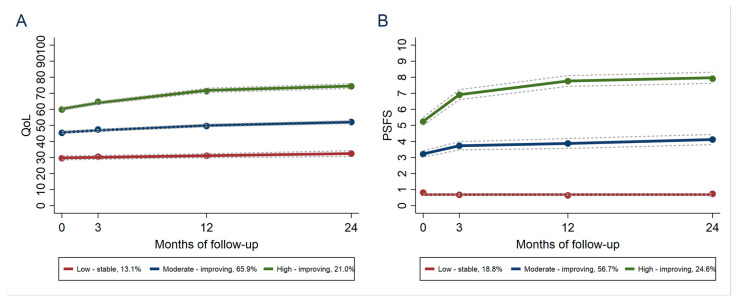
Group-based trajectories for (**A**) KOOS/HOOS quality of life and (**B**) Patient Specific Functional Scale. Each point represents the mean value for each trajectory. The solid line depicts the predicted trajectory, and the short-dashed lines represent 95% confidence intervals. The labels give the proportion of participants assigned to each trajectory. QOL = knee/hip-related quality of life; PSFS = Patient Specific Functional Scale.

Four distinct trajectories were identified for BMI (Figure 1): 31% were classified as normal weight, 43% as slightly overweight, 20% as overweight and 6% as obese. Over the two-year follow-up period, the four BMI trajectories only showed minimal changes (Figure 1), indicating that all four groups, irrespective of group affiliation, had a stable BMI. We identified three trajectory groups for both quality of life and activity limitations which were characterized as “low-stable”, “moderate-improving” and “high-improving” (Figure 2). For both outcomes, the trajectories were characterized by similar change patterns. The low-stable trajectory groups had low baseline scores with minimal improvement over the two-year follow-up period. The moderate trajectory groups showed some improvement, which indicates an improved quality of life and reduced activity limitation, whereas the high trajectory groups had a higher baseline score and considerable improvement from baseline to two years, especially for activity limitations.

The model selection parameters from the 2-stage model selection process are available in Table S1. The average posterior probability of group membership for each trajectory ranged from 0.83 to 0.96, indicating a good model fit (Table S2).

Table 2 shows the baseline characteristics for the four identified BMI trajectories. Age was significantly lowest in the obese group, and pain increased and general health (EQ5D, VAS) decreased with a higher BMI. The percentage of respondents with knee OA increased with increasing BMI, from 59% in the normal weight group to 80% in the obese group. The percentage of participants on sick leave was highest in the overweight and obese groups, with 25% and 26%, respectively. From the normal weight group to the obese group, pain increased from 4.9 (±1.8) to 6 (±1.9), and general health decreased from 69.1 (±17.4) to 53.3 (±19.1), respectively. Except for pain between the overweight and obese groups, the post hoc comparisons showed statistically significant (*p* < 0.001) differences between all BMI groups for the baseline variables.

Table 3 shows the probabilities of the two trajectory groups (quality of life and activity limitations) conditional on BMI group. The majority of all four BMI groups were linked to the moderate-improving group, both for quality of life (between 51.5% and 62.7%) and activity limitations (between 48.5% and 58.3%) (Table 3). A pronounced distinction was found for the proportion of patients linked to the low-stable group; 8.9% of the normal weight group compared to 35.9% of the obese group belonged to the low-stable quality of life group. For activity limitations, 16.8% of the normal weight group and 31.4% of the obese group were assigned to the low-stable group.

The sensitivity analysis including only participants with outcome data from three time points or more, *n* = 2794 for quality of life and *n* = 2639 for activity limitations, also revealed three trajectories (Figures S1 and S2). The shape of the curves and the percentage distribution within the three trajectory groups were almost identical to those identified in the primary analysis.

## 4. Discussion

The results from the present study revealed four distinct BMI trajectories, with minimal changes, irrespective of weight category, over the two-year follow-up period. There were highly significant differences between all four BMI groups for baseline variables; age decreased, pain increased and general health decreased with a higher BMI (Table 2). The majority of all four BMI groups were linked to the moderate or high improvement group, both for quality of life and activity limitations (Table 3).

The relative distribution of BMI categories showed that the normal weight and slightly overweight groups represented 31% and 43% of the total population, respectively. Changes over time showed a stable trajectory for all four groups over the two-year period (Figure 2). Radojčić et al. [27] identified BMI trajectories based on a cohort of postmenop ausal women followed over a 19-year period and found that slightly overweight women (BMI 25–27) had no different risks of pain or mortality than normal-weighted women. They suggested targeting a BMI below 27 might be an initial goal for weight reduction concerning musculoskeletal pain, as overweight to obese BMI patterns were mutually related to musculoskeletal pain. Their findings might be important information to give our future knee and hip OA patients, to motivate normal weight and slightly overweight groups to maintain a steady weight over the years to come.

We named the four BMI trajectories in accordance with Winter’s meta-analysis [28], which aimed at assessing all-cause mortality risk associated with BMI in those aged 65 or older in community-based populations. The authors concluded that precautions should be taken when applying the World Health Organization (WHO) healthy weight range for individuals 65 years and older and that a higher healthy weight range should be recommended. These suggestions were confirmed in a newly published paper from Visaria [29] who found a decreased all-cause mortality rate from a BMI of 25.0–29.9 in adults 65 years and older. Both studies indicate that applying WHO’s reference BMI categories for overweight might be misleading in older adults. The results from the present study indicate that also expanding the healthy weight range for OA patients should be plausible. The quality of life and activity limitation group memberships conditional on BMI showed that more than 80% percent of the slightly overweight group showed moderate-to-considerable improvements both for quality of life and activity limitations (Table 3). Importantly, these results indicate that being slightly overweight does not preclude improvement in important patient-reported outcomes.

In a recent systematic review, Solanki et al. [13] found that weight gain was associated with the worsening of clinical and structural features in knee OA. Based on previous studies showing that weight loss interventions have a limited impact on reducing pain and disability, they suggest that weight gain prevention may be considered a more feasible and realistic strategy than weight reduction to improve outcomes in knee OA patients. The four BMI groups identified in the present study (Figure 1) all showed a stable BMI over time. Even if the obese BMI group showed the smallest improvements (Table 3), the majority of all four BMI groups showed moderate-to-high improvements considering both quality of life and activity limitations. Our findings support Solanki’s suggestion that a weight-neutral approach focusing on exercises and physical activities should be the cornerstones in the treatment of mild and moderate OA.

We identified three distinct trajectory groups both for quality of life (Figure 2A) and activity limitations (Figure 2B). The high-improving trajectories, which also showed the highest scores at baseline, showed considerable improvement during the intervention period (up to three months) and the curves continued to show an increasing tendency up to two years. The low-stable groups were characterized by low baseline scores and showed minimal fluctuations over the study period. The results are in line with the function trajectories identified by Lee et al. [30], where patients with initial higher functional scores showed an early improvement compared to patients with low initial scores during a twelve-week intervention program. This might indicate that the implementation program is better adapted to those with better function when they contact health care providers, and that patients with low baseline scores should be offered treatment tailored to the patient’s potentially more complicated clinical needs and a closer follow-up.

In contrast to the findings from the present study, a recent systematic review from Holden et al. [16] identified two moderators for reducing pain and improving physical function. Patients reporting the most severe pain and the poorest physical function at baseline generally benefited the most from exercise programs, with the evidence most certain in the short run. No other treatment effect modifiers (like age, obesity or comorbidities) were identified. In the discussion, they raise uncertainty about the role of exercise in osteoarthritis and that improvements might primarily be driven by placebo, contextual factors and the natural course of the disease. The present analyses of register data show that respondents with initial higher scores and belonging to the two lowest BMI categories showed the highest improvements both for quality of life and activity limitations. However, the majority of all respondents were linked to the moderate or high improving group, both for quality of life (between 51.5% and 62.7%) and activity limitations (between 48.5% and 58.3%) (Table 3), indicating that regardless of baseline characteristics, significant long-term improvements might be expected. Unlike the findings in the review [16] showing small effects in the medium (6 months) and long term, (12 months), the results from the present study state that the improvements did not decline but stayed stable over the two-year period. One explanation might be that the AktivA model includes both an educational self-management part, the osteoarthritis school, and the possibility of future follow-ups. Giving the patients tools for managing the condition on their own but knowing that they have the opportunity for a booster session now and then might be crucial for long-term success.

In line with many register and large population-based studies, the patients in our study self-reported their body weight and height. People tend to underestimate their weight and overestimate their height, which must be considered when interpreting our results. Particularly in the setting of case–control studies, differential perceptions of the possible negative influence of being overweight on health or disease development, could result in a recall bias [31]. However, Yoong et al. [32] investigated the agreement between self-reported and measured height and weight in general practice patients. They found that informing the participants that their height and weight would be directly measured after providing their self-reported height and weight did not change the accuracy of the self-report. They found an overall agreement between measurement methods of 80% and concluded that in large surveillance studies, self-report is likely to be an accurate alternative. In the AktivA register, the patients reported height and weight at baseline and additionally reported weight at 3, 12 and 24 months. When they received the next follow-up questionnaires, their previously reported weight numbers were hidden.

In the present study, the response rates decreased during the study period, with a response rate of 84%, 61% and 53% at 3, 12 and 24 months, respectively. As the participants received three online follow-up questionnaires over the two-year study period, we expected the response rate to decline considerably. In a systematic review, Meyer et al. [33] found that response rates at one single time point were 46% and 51% for web-based and email surveys, respectively. A meta-analysis concluded that the average online response rate was 44%, and that sending surveys to a clearly defined population positively impacted the survey response rate [34]. Compared to these reviews, the AktivA participants’ response rates, including four follow-up contacts, are satisfactory and indicate that the participants are still positive to share their experiences and outcomes up to two years after inclusion.

Over the last decade, group-based trajectory modeling has been frequently used to identify subgroups of individuals participating in longitudinal cohort studies. The statistical method provides the researcher with results presented in the form of figuratively depicted curves (trajectories), which can easily be understood by readers of scientific papers and accessible to nontechnical audiences [24]. However, in an editorial, Thomas discussed limitations that the group-based trajectory modeling approach might have on identifying distinct subgroups [35]. He claimed that the long periods between follow-ups may not reflect people’s overall symptoms over time or symptom fluctuations among participants within different trajectories [35]. When excluding participants with data from only two or less time points, the shape of the curves and the percentage distribution within the three trajectory groups were almost identical to those identified in the primary analysis. Therefore, we do not consider the missingness of participants who lacked responses to have any impact on the main results from the primary analysis.

Register studies lack a control group; however, the high number of included participants should still provide generalizability and evidence of the effectiveness of the treatment. Over the two-year period, the response rate was declining, from 84% at 3 months to 53% at 24 months. In surveys, there is often a problem that the number of non-responders exceeds the number of responders. However, in the present study, the relatively high response rate even after two years reduces the likelihood of non-response bias.

## 5. Conclusions

Four distinct BMI trajectories, which all showed minimal changes over time, were identified. Almost 70% of the participants belonged to the slightly overweight, overweight and obese groups. Despite no significant weight reduction over the two-year period, 8 in every 10 participants improved their quality of life (87%) and reduced their activity limitations (81%) after participating in the AktivA program. The study highlights the importance of introducing patients with OA to an exercise and self-management program and that a focus on BMI and weight loss should be less emphasized.

## Figures and Tables

**Table 1:** Baseline characteristics of the study sample.

	Total Population N = 4265
Age (SD ^1^)	63 (9.4)
Female (%)	74
Body weight, kg (SD)	82.2 (16.2)
BMI ^2^ (kg/m^2^)	28.1 (4.9)
Hip/Knee OA (%)	32/68
Sick leave (%)	18
Pain NRS ^3^ (0–10)	5.2 (1.8)
General health (EQ-5D VAS ^4^)	64.4 (18.6)

^1^ SD: standard deviation, ^2^ BMI: body mass index, ^3^ NRS; numeric rating scale, ^4^ EQ-5D 5L VAS (Euro QoL 5L, health-related quality of life, visual analogue scale).

**Table 2:** Baseline patient characteristics for the four BMI groups.

	Normal Weight	Slightly Overweight	Overweight	Obese	*p*-Value
N (%)	1327 (31)	1830 (43)	866 (20)	242 (6)	
Age (SD ^1^)	64.9 (9.7)	63.4 (9.1)	61.4 (9.1)	57.7 (8.9)	<0.001
Female%	80	68	76	76	
Body weight, kg (SD)	67.4 (8.4)	82.2 (9.8)	95.7 (10.5)	115 (13.9)	<0.001
BMI ^2^ (kg/m^2^)	23.1 (1.7)	27.8 (1.6)	32.9 (1.8)	39.8 (3.2)	<0.001
Hip/Knee%	41/59	30/70	25/75	20/80	
Sick leave%	12	16	26	25	
Pain (NRS ^3^ 0–10)	4.9 (1.8)	5.2 (1.8)	5.6 (1.8)	6 (1.9)	<0.001
General health (EQ-5D VAS ^4^)	69.1 (17.4)	65.1 (18.2)	59 (18.5)	53.3 (19.1)	<0.001

^1^ SD: standard deviation, ^2^ BMI: body mass index, ^3^ NRS; numeric rating scale, ^4^ EQ-5D VAS; European Quality of Life-five dimensions Visual analogue scale (0–100).

**Table 3:** Probabilities of (A) KOOS/HOOS quality of life group membership conditional on BMI group membership and (B) PSFS group membership conditional on BMI group membership.

	A: QoL ^1^ Group		
BMI ^2^ Group	Low-Stable	Moderate-Improving	High-Improving
Normal weight	8.9	61.2	29.9
Slightly overweight	13.1	62.7	24.3
Overweight	24.8	62.3	12.9
Obese	35.9	51.5	12.6
		B: PSFS ^3^ Group	
BMI Group	Low-Stable	Moderate-Improving	High-Improving
Normal weight	16.8	48.5	34.7
Slightly overweight	18.2	54.6	27.2
Overweight	22.2	58.3	19.5
Obese	31.4	57.0	11.6

^1^ QoL, quality of life,; ^2^ BMI, body mass index; ^3^ PSFS, Patient Specific Function Scale.

## Data Availability

Data will not be shared at this stage. Data from the present study are extracted as a part of a large, ongoing national quality register and several analyses are planned in the future.

## Supplementary Materials

The following supporting information can be downloaded at: https://www.mdpi.com/article/10.3390/jcm12227094/s1, Table S1: The Bayesian information criteria (BIC) values for group-based trajectory modelling according to the number of trajectory group; Table S2: Fit indices of selected models for body mass index, KOOS/HOOS quality of Life, and patient-specific functional scale; Figure S1: Group-based trajectories, including only participants with outcome data from three time points or more for body mass index. Each point represents the mean value for each trajectory. The solid line depicts the predicted trajectory, and the short-dashed lines represent 95% confidence intervals. The labels give the proportion of participants assigned to each trajectory; Figure S2: Group-based trajectories, including only participants with outcome data from three time points or more for (A) KOOS/HOOS quality of life and (B) Patient Specific Functional Scale. Each point represents the mean value for each trajectory. The solid line depicts the predicted trajectory, and the short-dashed lines represent 95% confidence intervals. The labels give the proportion of participants assigned to each trajectory. QOL = knee/hip-related quality of life; PSFS = Patient Specific Functional Scale.
