# Supplementary material for: Body Mass Index, Quality of Life and Activity Limitation Trajectories over 2 Years in Patients with Knee or Hip Osteoarthritis: A Dual Trajectory Approach Based on 4265 Patients Included in the AktivA Quality Register
Source: J Clin Med. 2023 Nov 14;12(22):7094. doi: 10.3390/jcm12227094 (PMC10672133; doi:10.3390/jcm12227094)
Supplement: Supplementary file 1 [file jcm-12-07094-s001.zip › jcm-2672542-supplementary.pdf]

Supplementary material

Table S1. The Bayesian information criteria (BIC) values for group-based trajectory modelling according to the number of trajectories groups.

Outcome	Number of groups	BIC[a]	BIC[b]
Body mass index		n=4,265	n=12,546
	2	-33,774	-33,779
	3	-31,631	-31,637
	4	-30,075	-30,084
	**4 optimized[d]**	**-30,063**	**-30,069**
KOOS/HOOS QOL		n=4,265	n=13,014
	2	-53,348	-53,352
	3	-52,931	-52,938
	4[c]	-52,834	-52,843
	**3 optimized[d]**	**-52,927**	**-52,933**
PSFS		n=4,265	n=12,523
	2	-28,142	-28,146
	3	-27,621	-27,628
	4[c]	-27,439	-27,447
	**3 optimized[d]**	**-27,582**	**-27,589**

KOOS=Knee injury and Osteoarthritis Outcome Score; HOOS=Hip disability and Osteoarthritis Outcome Score; QOL=Knee/Hip-related quality of life; PSFS=Patient Specific Functional Scale; [1] BIC = Bayesian information criterion (for the total number of participants); [2] BIC = Bayesian information criterion (for the total number of observations); [a] One or more groups had <5% of the participants in the sample; [d]The final model was optimized by removing non-significant polynomial terms

Table S2. Fit indices of selected models for body mass index, KOOS/HOOS Quality of Life, and patient-specific functional scale

Outcome	Average posterior probability	Estimated group probability	Percentage assigned[a]
**Body mass index**			
Normal weight	0.94	31.1	31.1
Slightly overweight	0.93	42.6	42.9
Overweight	0.94	20.6	20.3
Obese	0.96	5.6	5.7
**KOOS/HOOS QOL**			
Low - stable	0.83	15.5	13.1
Moderate - improving	0.86	61.5	65.9
High - improving	0.86	23.0	21.0
**PSFS**			
Low - stable	0.86	19.3	18.8
Moderate - improving	0.83	53.6	56.7
High - improving	0.84	27.1	24.6

KOOS=Knee injury and Osteoarthritis Outcome Score; HOOS=Hip disability and Osteoarthritis Outcome Score; QOL=Knee/Hip-related quality of life; PSFS=Patient Specific Functional Scale; [a] Percentage of participants assigned according to the maximum posterior assignment rule.

An average posterior probability exceeding a minimum threshold of 0.7 for each group is suggested to indicate high assignment accuracy. There should be a close correspondence between the estimated group probability and the percentage of participants assigned according to the maximum posterior assignment rule.

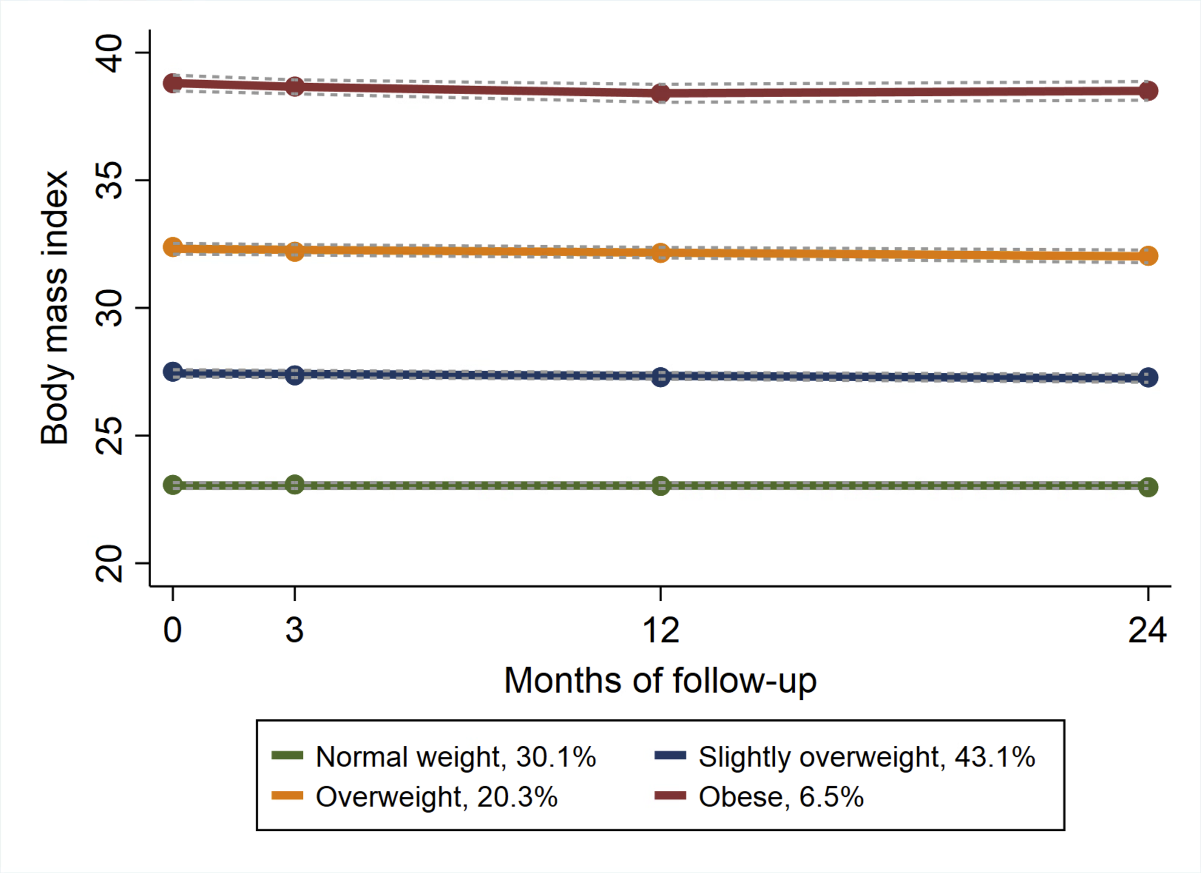

Figure S1. Group-based trajectories, including only participants with outcome data from three time points or more for Body mass index. Each point represents the mean value for each trajectory. The solid line depicts the predicted trajectory, and the short-dashed lines represent 95% confidence intervals. The labels give the proportion of participants assigned to each trajectory.

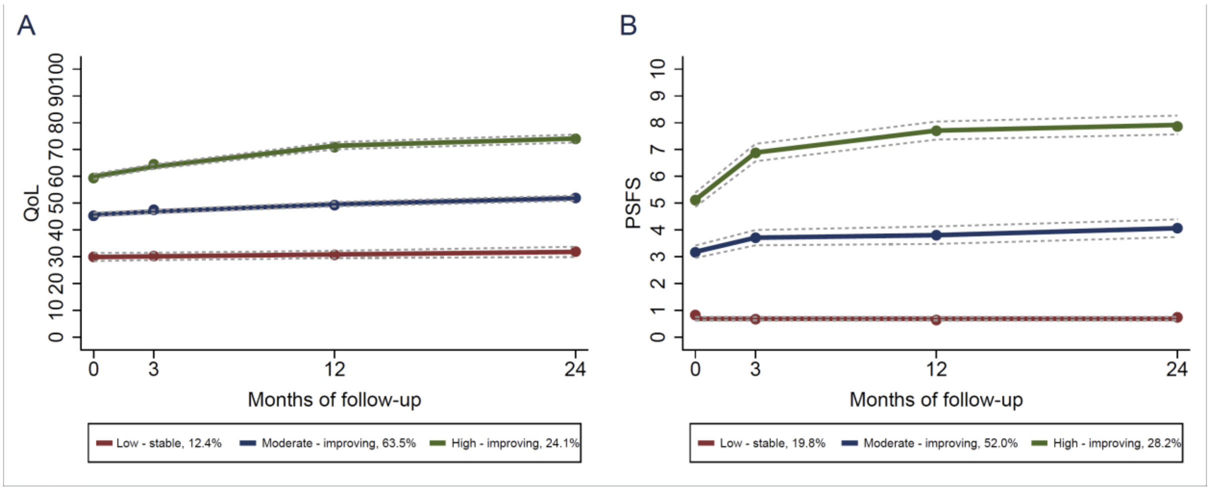

Figure S2. Group-based trajectories, including only participants with outcome data from three time points or more for (A) KOOS/HOOS quality of life and (B) Patient Specific Functional Scale. Each point represents the mean value for each trajectory. The solid line depicts the predicted trajectory, and the short-dashed lines represent 95% confidence intervals. The labels give the proportion of participants assigned to each trajectory. QOL=Knee/Hip-related quality of life; PSFS=Patient Specific Functional Scale
